# The ethical, legal and social issues of neuroergonomics in the workplace

**DOI:** 10.3389/fnrgo.2026.1923291

**Published:** 2026-08-26

**Authors:** Sherry I. Brandt-Rauf, Paul W. Brandt-Rauf

**Affiliations:** 1 Department of Environmental and Occupational Health Sciences, Dornsife School of Public Health, Drexel University, Philadelphia, PA, United States; 2 School of Biomedical Engineering, Science and Health Systems, Drexel University, Philadelphia, PA, United States

**Keywords:** ethical, legal, neuroergonomics, social, workplace

## Abstract

In the near future it is anticipated that neuroergonomic technologies will be widely deployed in multiple real-world applications including many work environments. Neuroergonomics will be especially relevant in an increasingly knowledge-based, technology-driven economy where there will likely be significant incentives to apply it to increase worker productivity. However, this accelerating convergence of the brain, technology and work also raises serious issues including the privacy rights of workers' neural data and the protection of workers from coercion and discrimination over the use of the technology. Current ethical, legal and social frameworks are inadequate and unprepared to optimally deal with these issues which are rapidly being outpaced by the technological advances.

## Introduction: Innovation

Advances in neurotechnology have made great strides in improving the treatment of certain neurological conditions, but, until recently, these techniques have been highly invasive, risky and applicable only in controlled experimental clinical situations. However, neuroergonomics has adapted these techniques to make them noninvasive, largely safe, miniaturized, wearable, and portable so that they can be used in real-world environments, including the workplace. Neuroergonomics is thus now capable of monitoring and altering cognitive function at work in real-time to ensure and sustain optimal neural efficiency during the performance of a variety of workplace tasks. Neuroergonomics can improve safety/security, enhance human/computer/robot interactions, expedite training/skill acquisition, facilitate neurorehabilitation, and generally promote worker neurocognitive enhancement. In an economy that is becoming increasingly knowledge-based and reliant on optimal technology utilization, it can be anticipated that neuroergonomics will be increasingly relied upon to maintain and promote workplace productivity (Brandt-Rauf and Ayaz, 2024).

The two critical components of neuroergonomics are the technology for monitoring brain activity and the technology for altering brain activity. The most significant advance in the former has been the introduction of functional near infrared spectroscopy (fNIRS) for portable neuroimaging of brain activity used either alone or in conjunction with other physiologic measurements (such as electroencephalography, pupillometry and dermal conductivity). fNIRS can assess cognitive effort in specific brain regions by analyzing the changes in oxygenated and deoxygenated hemoglobin via infrared spectroscopy. This cognitive effort, when coupled with behavioral task measures such as task accuracy and/or task duration, can be shown to represent a comprehensive measure of neural efficiency (NE), i.e., the difference between the behavioral efficiency of task performance (P) and the cognitive effort of brain activation (CE) (Curtin and Ayaz, 2019). The most significant advance in altering brain activity has been the introduction of portable devices for transcranial direct brain stimulation with electrical currents (tDCS) or magnetic fields (TMS) (Woods et al., 2016).

Finally, neuroimaging can be used together with neurostimulation in a closed-loop system (Esmaielpour et al., 2020). For example, neuroimaging can identify the area of the brain that needs to be targeted, neurostimulation can be directed to that specific area, and further neuroimaging can be used to monitor the effect until the neurostimulation achieves the optimal state of neural efficiency.

## Applications: Business

Neuroergonomics has potential applications in many fields. As expected, initially it has been investigated for the prevention and treatment of a variety of brain pathologies, including neurological and psychological diseases, neurodevelopmental and neurodegenerative disorders, and brain trauma, with some success (Gonsalvez et al., 2021; Antal et al., 2026). However, very rapidly its use has been extended to investigations of brain function in normal, healthy subjects involved in many different activities, including in education and training (Martin et al., 2014; Brockington et al., 2018; Cox et al., 2020; Tan et al., 2023; Shi et al., 2023; Li et al., 2024; Song et al., 2025), military operations (Feltman et al., 2020; Lu et al., 2022; Nadler et al., 2025), legal practice (Bhutta et al., 2024; Wang et al., 2024), and athletic endeavors (Colzato et al., 2017; Kamali et al., 2023; Chmiel and Kurpas, 2026), among others, once again with some success.

Another place where neuroergonomics in being investigated is in business, for example to improve product development and marketing (Krampe et al., 2018; Liu et al., 2018; Gier et al., 2020; Balconi et al., 2023; Oishi et al., 2023). However, it is anticipated to have its most significant business impacts in the workplace itself. For example, neuroergonomics can contribute to providing better accommodations for neurodivergent workers (Albuquerque and Ribeiro, 2025). Neuroergonomics can also be used to provide better workplace design for all workers. For example, fNIRS studies have shown positive effects of ergonomic workstations vs. nonergonomic workstations correlating with improved task performance (Alyan et al., 2021a,b). On the other hand, an fNIRS study of active workstations showed that cognitive resources were reallocated during bicycling at an active workstation indicating a competition for these limited resources when multitasking (Huang et al., 2019). In addition, neuroergonomics may improve the assessment of cognitive readiness for employees with depressive symptoms returning to work (Atsumori et al., 2019).

fNIRS has obvious applications for monitoring neural efficiency in safety-sensitive or security-sensitive jobs. For example, an experimental study of subjects engaged in a sustained attention reaction time task showed significant differences between the first ten and last ten minutes demonstrating the potential use for tasks that require sustained attention but subject to compromise by distraction or multitasking (Derosiere et al., 2014). Similarly, subjects engaged in an auditory task under different physical activity situations, such as sitting, walking indoors or walking outdoors, suggested that work involving memory could be compromised by simultaneous physical activity (McKendrick et al., 2017). fNIRS has also been used to evaluate workers' responses in simulated training environments. A study of pilots in a flight simulator vs. a real aircraft showed that pilots in the aircraft had higher cortical activation leading to more errors than those in the simulator during cognitively demanding tasks (Gateau et al., 2018). An fNIRS study of mental workload in urban transit drivers under simulated conditions suggested it could help companies develop reasonable shiftwork schedules and ensure operator safety (Li et al., 2019). Furthermore, an fNIRS study of coal mine workers under actual work conditions provides additional support for this by demonstrating significant differences in cognitive ability before and after work shifts (Tian et al., 2022). Finally, with the increasing use of robots in the workplace, it becomes increasingly important to optimize robot-human interactions. An fNIRS study of the neural correlates of emotional processing of human and robot stimuli indicated how this might enable optimization of social robot design (Yorgancigil et al., 2022).

Like fNIRS, transcranial brain stimulation is likely to have significant impacts on the workplace by augmenting human performance during complex tasks (McKinley et al., 2012). For example, tDCS during a vigilance task caused significant improvement in target detection demonstrating its ability to decrease performance degradation during sustained attention tasks (Nelson et al., 2014). Similarly, in a real-world work environment of sleep-deprived nurses, TMS was used to modulate the sustained attention and working memory after a night shift and significantly reduced mental fatigue (Wang et al., 2026). tDCS can also augment training for cognitive tasks. For example, when applied during skill acquisition, tDCS led to immediate improvements in performance that even persisted the following day (Martin et al., 2014). tDCS can also significantly improve motor learning and fine motor task performance even in very highly skilled tasks such as robotic surgery (Patel et al., 2022; Wilson et al., 2022).

Finally, as noted, neuroimaging can be used in conjunction with neurostimulation in a closed-loop system allowing for direct monitoring of interventions to optimize the effects (Curtin et al., 2019b; Patel et al., 2020). For example, one study employed fNIRS to monitor tDCS-linked improvements in working memory of participants (Jones et al., 2015). Another study, used fNIRS to monitor brain activity of participants who received repetitive TMS during a speed of processing task to demonstrate significantly enhanced neural efficiency (Curtin et al., 2019a).

## Issues: Ethical, legal and social

We are already beginning to see neuroergonomics move from the experimental to the applied, and it has been projected to be as common as computers and cell phones in coming decades (Gaudry et al., 2021). So questions arise as to how this should be used and who should decide. In the workplace, it seems likely that employers will see significant reason to induce workers, overtly or covertly, to augment their cognitive capacity through neuroergonomics to increase workplace productivity (Farah et al., 2004), since some employers already offer workers enhancements to their capabilities through chemical (e.g., caffeine) or mechanical (e.g., computer-based augmentation) means (Gaudry et al., 2021). Usage of neuroergonomics could become a specific job requirement, and employers could even offer contracts specifying working hours based on measured cognitive workload rather than standard working hours (Acarturk and Mucen, 2022). Discrimination by employers and insurers against workers who refuse to comply may become a real threat (Appel, 2008).

Even without employer coercion, workers may feel compelled to pursue neuroenhancement on their own in order to compete with enhanced co-workers, autonomous robots, or artificial general intelligence agents. For example, we already know that students have used chemical stimulation for years to enhance performance, and that now a large proportion (>90% in a survey of Swiss college students) are prepared to try new products that increase IQ (Ott and Biller-Andorno, 2014). So it is not surprising that a community of do-it-yourself brain stimulators already exists with blogs and websites dedicated exclusively to the topic; the most active tDCS forum on Reddit averages several posts per day, and the two most popular tDCS tutorial videos on YouTube have garnered tens of thousands of views each (Wexler, 2016). Although neuroergonomics experts have expressed concern about these issues, the general public may be more accepting. For example, a survey of people in Japan found that approximately 20% of respondents stated that they were willing to use enhancement technologies and 80% were tolerant toward others' use so that “there was no need to ban such technologies” (Nakazawa et al., 2022). On the other hand, neuroscientists themselves are less inclined to use these technologies on themselves. For example, a survey of researchers found that relatively few (8%) had used brain stimulation on themselves for neuroenhancement due to safety and other concerns, even though a substantial number (39%) considered it to be potentially useful for real-life application in healthy populations (Shirota et al., 2014). However, in general, most experts are alarmed about do-it-yourself brain stimulation for a long list of reasons: off-target brain effects; interaction with ongoing brain activity; increasing some abilities while potentially decreasing others; effects lasting too long; small changes leading to bigger effects; interindividual variability; risk/benefit ratio (Wurzman et al., 2016).

## Conclusion: The future of policy and practice

Given the above considerations, when it comes to the use of neuroergonomics technologies it seems logical to ask: Should we/can we protect employees from employers? Should we/can we protect employees from themselves?

One important aspect of the first question is who should have access to data obtained from monitoring of brain activity. As has been pointed out, such data may not be considered medical or health data whose privacy would be covered by existing legislation (Gaudry et al., 2021). An analogy has been made to genetic data where legal protections have been enacted, and the fact that connectome may be even more sensitive data since it is a more immediate and intimate representation of the individual than their genome (Gaudry et al., 2021). In response in the U.S., fours states have recently included neural data in their consumer privacy data laws. A federal law has also been proposed to establish a framework to protect neural data against privacy risks, including risks of discrimination in the workplace such as might arise in “neuroergonomic” uses that assess worker productivity (Libin et al., 2025). The European Union currently lacks specific regulations or directives dedicated to neurotechnologies, but protections have been proposed, and a few countries have implemented frameworks specifically designed to protect individual rights in this context (EBC, 2025). A second aspect of this question relates to the coercive abuse of neurotechnology by employers not just for monitoring but also for enhancement. Once again, the necessity for legal remedies has been suggested (Gaudry et al., 2021), but much less attention has been paid to this issue. The aforementioned proposed federal legislation does note that neurotechnology includes devices that stimulate or alter the nervous system to influence its function and that guidance it needed to ensure ethical use of neurotechnology with transparency and opt-in mechanisms (Libin et al., 2025).

The second question may be equally difficult to address, especially with regard to do-it-yourself enhancement which is likely to accelerate as products become more commercially available. It has been suggested that we may need to require manufacturers of neurotechnology devices to disclose to users appropriate usage procedures and all possible side effects despite that they are not yet fully known (Gaudry et al., 2021). Currently, however, it appears that these devices may not fall under the scope of FDA regulation (Antal et al., 2026). Once again, the proposed federal legislative framework may eventually lead to guidance on these issues if we can afford to wait (Libin et al., 2025). In the workplace, there will be an important role for occupational health professionals to advise employees and employers on the ethically appropriate usage of neural data and neural devices (Brandt-Rauf and Ayaz, 2024).

The overarching issue is whether it is right to pursue human enhancement at all, in the workplace or elsewhere and of the brain or otherwise. There are reasonable arguments both for and against enhancement. Anti-enhancement arguments tend to put great weight on the unnatural threat to the human essence (Wagner et al., 2019). Pro-enhancement arguments tend to rely on the autonomous right of the individual to self-improvement (Harris, 2007). One particularly intriguing aspect of the latter stems from the apparent ability of neural technologies for moral enhancement, including increasing altruism, trust, cooperation, fairness, empathy and a willingness to help others (Darby and Pascual-Leone, 2017). So neural enhancement might make us less human but more humane. In the end, as with all technologies, it is not that they are good or bad in themselves, but rather how we choose to use them that makes the difference.

## Data Availability

The original contributions presented in the study are included in the article/supplementary material, further inquiries can be directed to the corresponding author.
